# Supplementary figures and images for: Aberrant Expression and Prognostic Potential of IL-37 in Human Lung Adenocarcinoma
Source: Biomedicines. 2022 Nov 24;10(12):3037. doi: 10.3390/biomedicines10123037 (PMC9775426; doi:10.3390/biomedicines10123037)

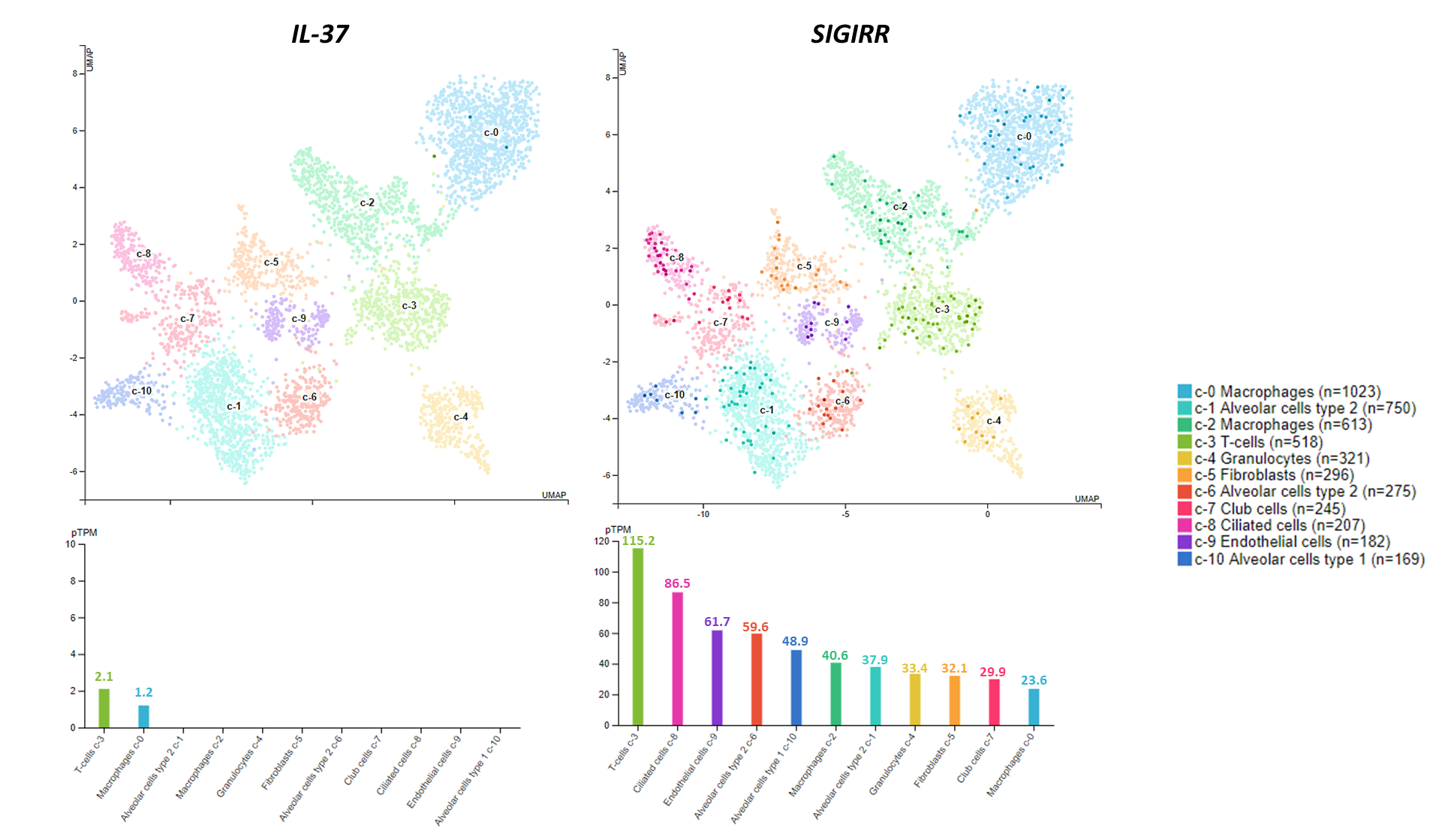

Supplement: Supplementary file 1 [file biomedicines-10-03037-s001.zip › Christodoulou et al_Suppl Figure S1.tif]

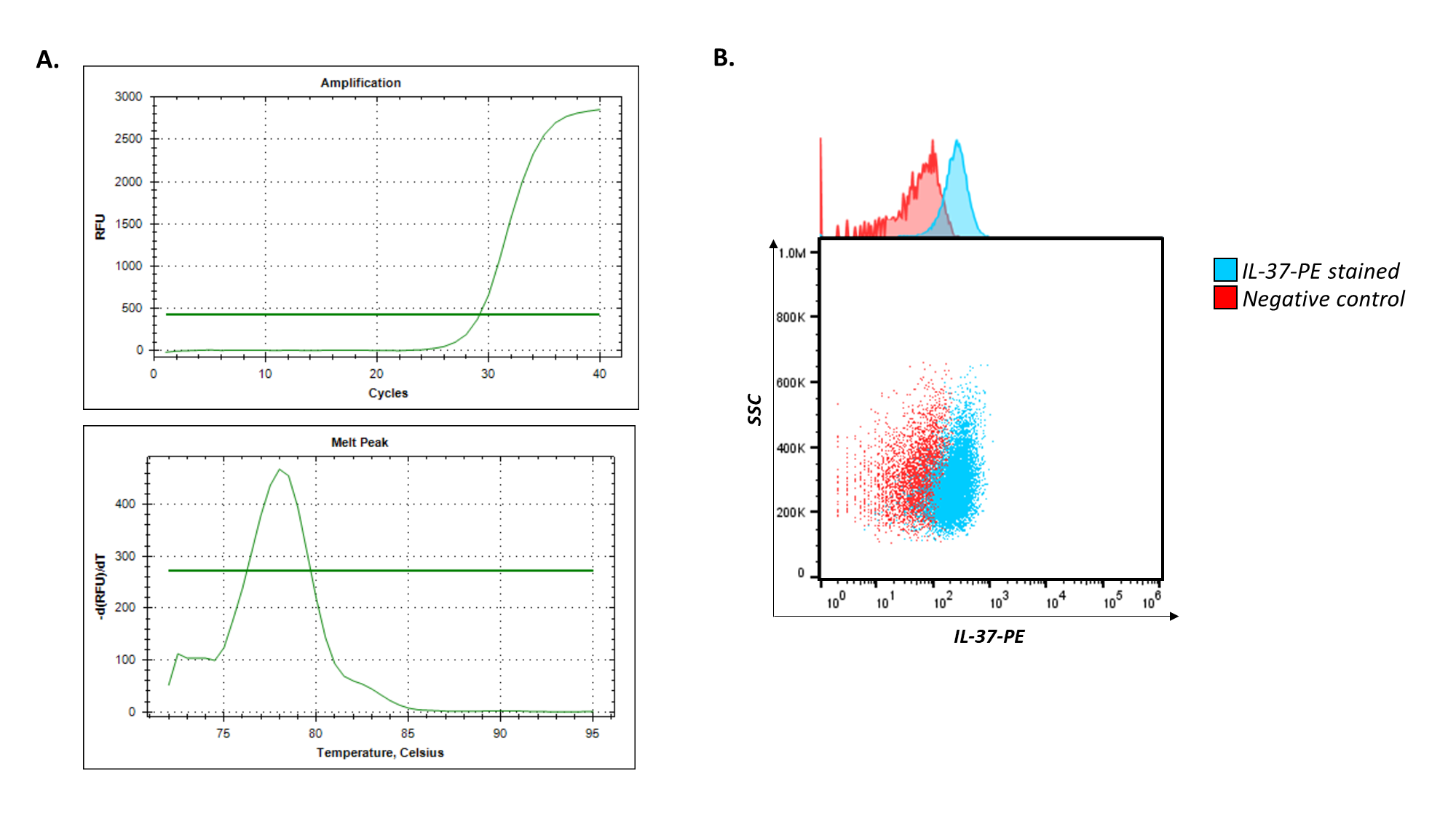

Supplement: Supplementary file 1 [file biomedicines-10-03037-s001.zip › Christodoulou et al_Suppl Figure S2.tif]
